# Leptomeningeal Carcinomatosis: A Rare Complication of Metastatic Gastric Cancer

**DOI:** 10.7759/cureus.34201

**Published:** 2023-01-25

**Authors:** Steven Y Lai, Sharonlin Bhardwaj, Phillis Wu

**Affiliations:** 1 Internal Medicine, Olive View University of California Los Angeles Medical Center, Sylmar, USA; 2 Hematology and Oncology, Olive View University of California Los Angeles Medical Center, Sylmar, USA

**Keywords:** intrathecal methotrexate, metastatic gastric cancer, central nervous system diseases, leptomeningeal metastasis, leptomeningeal disease

## Abstract

Leptomeningeal disease, also known as leptomeningeal carcinomatosis, occurs when cancer metastasizes to the meninges. This rare complication is associated with a poor prognosis. It is most commonly seen in patients with metastatic breast cancer, lung cancer, and melanoma. However, it is extremely rare in patients with metastatic gastric cancer.

A 64-year-old female with poorly differentiated gastric adenocarcinoma metastatic to the peritoneum developed new neurological symptoms twelve months after initiating palliative chemotherapy. Her uptrending tumor markers, brain magnetic resonance imaging (MRI) findings, and lumbar puncture results were consistent with leptomeningeal disease. The patient was started on treatment with intrathecal methotrexate (IT MTX), which resulted in significant improvement in her neurological symptoms.

Leptomeningeal disease in gastric cancer has limited treatment options due to poor blood-brain barrier penetration. IT MTX is a potentially effective treatment for patients with leptomeningeal disease from gastric cancer.

## Introduction

Leptomeningeal disease, also known as leptomeningeal carcinomatosis, is a rare complication of cancer involving metastatic disease in the central nervous system (CNS). This is typically seen in patients with metastatic breast cancer, lung cancer, and melanoma. However, it is extremely rare in patients with gastric cancer [1]. As a result, there is no established standard of care for leptomeningeal disease from gastric cancer [2]. In this report, we present a rare case of leptomeningeal disease in a patient with metastatic gastric cancer who responded well to treatment with intrathecal methotrexate (IT MTX).

## Case presentation

A 64-year-old Hispanic female with 15-pack-year smoking history and no other medical history presented to the emergency department complaining of abdominal pain. Vitals were unremarkable. The physical exam was notable for epigastric and right upper quadrant abdominal tenderness. Complete blood count (CBC) and comprehensive metabolic panel (CMP) were negative. A computerized tomography scan (CT) of the abdomen showed gastric thickening, and subsequent esophagogastroduodenoscopy (EGD) revealed enlarged gastric folds with friable mucosa. Biopsy confirmed poorly differentiated gastric adenocarcinoma without evidence of microsatellite instability. Human epidermal growth factor receptor 2 (HER2) was negative by fluorescence in-situ hybridization (FISH), and programmed cell death ligand-1 combined positive score (PD-L1 CPS) was <1. Carcinoembryonic antigen (CEA) at the time of diagnosis was elevated at 29.4 ng/mL (reference range <2.5 ng/mL), and carbohydrate antigen 19-9 (CA 19-9) was 1058 units/mL (reference range <35 units/mL). Diagnostic laparotomy for staging showed multiple small peritoneal nodules in the pelvis, consistent with metastatic gastric adenocarcinoma.

Over the next twelve months, the patient started treatment with two cycles of 5-fluorouracil, oxaliplatin, and leucovorin (mFOLFOX) due to her initial inability to tolerate pills. After her oral intake improved, she was then switched to ten cycles of capecitabine and oxaliplatin (XELOX). CEA decreased to a nadir of 7.5 ng/mL during cycle eight. Oxaliplatin was then discontinued due to cumulative toxicity and neuropathy, and she subsequently completed six cycles of capecitabine monotherapy. After a total of eighteen cycles, the patient developed new symptoms of headache, dizziness, nausea, and vomiting. CEA had increased to 31.9 ng/mL, and CA 19-9 had risen to 7300 units/mL. A computed tomography (CT) of the chest, abdomen, and pelvis showed persistent thickening of the gastric lining but no other sites of disease. Positron emission tomography/computed tomography (PET/CT) of the whole body and brain magnetic resonance imaging (MRI) were unrevealing.

**Figure 1:**
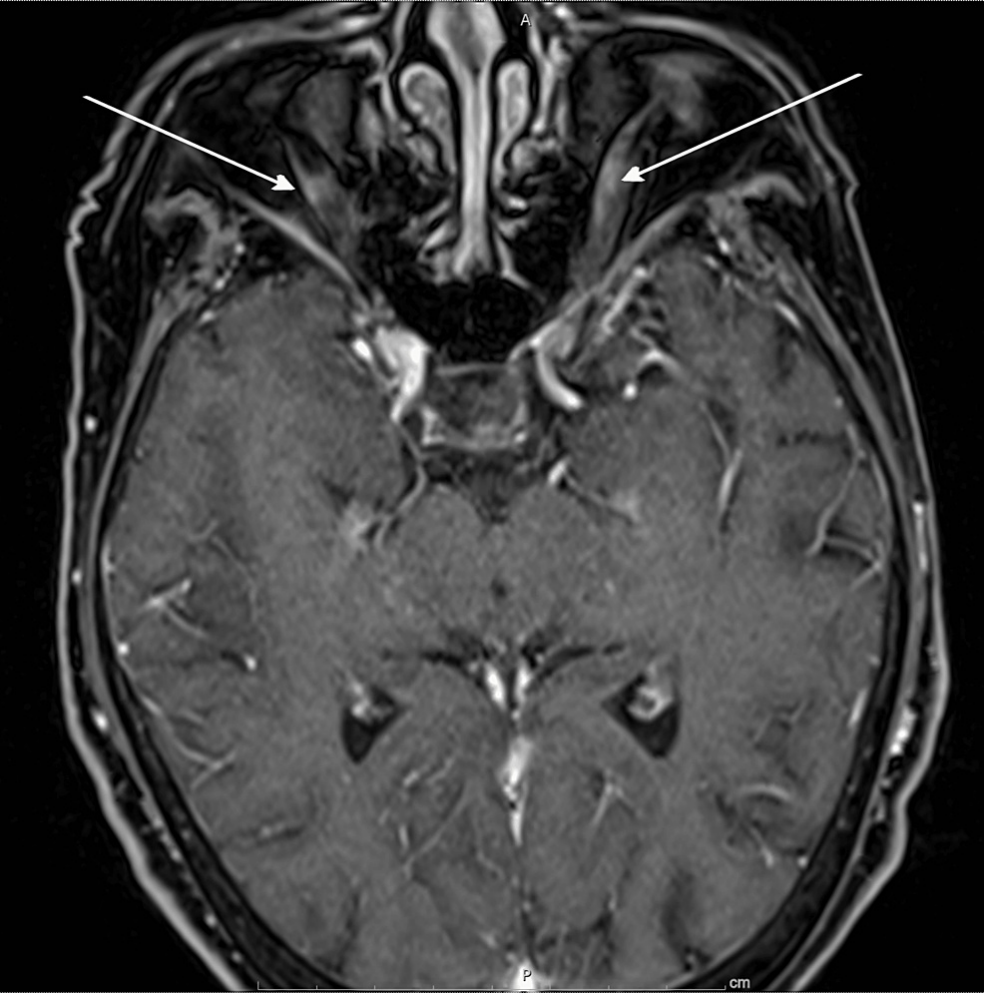
Patient’s head MRI with abnormal enhancement of optic nerves. MRI: magnetic resonance imaging.

During the next three weeks, the patient continued to complain of worsening headache and dizziness, along with new symptoms of visual changes and hearing loss. The exam was notable for decreased left-eye visual acuity, left-sided hearing loss, and ataxia. She was admitted for further evaluation. A repeat brain MRI showed abnormal enhancement along bilateral optic nerve sheathes (see Figure 1). Lumbar puncture (LP) was notable for an elevated opening pressure of >55 cm H_2_O (reference range 10-25 cm H_2_O). Cerebrospinal fluid (CSF) studies showed xanthochromia, with 175 red blood cells/mm^3^ (reference range 0-5/mm^3^), five nucleated cells/mm^3^ (reference range 0-5/mm^3^), decreased glucose of 35 mg/dL (reference range 40-70 mg/dL), and elevated protein of 54 mg/dL (reference range 15-45 mg/dL). The cytology was acellular. CSF tuberculosis polymerase chain reaction (PCR), coccidioides antibody, and cryptococcus antigen were all negative. CSF was also sent for a paraneoplastic panel. Oral dexamethasone, 4 mg daily, was initiated, which resulted in the improvement of her neurological symptoms. She was subsequently discharged home, with the result of the paraneoplastic panel still pending.

Two weeks later, the patient was hospitalized again due to new-onset seizures occurring multiple times per day. A repeat LP showed an elevated opening pressure of 48 cm H_2_O. Cytology showed atypical epithelioid cells but was negative for malignant cells. The results of the previously obtained paraneoplastic panel were negative. Based on the atypical epithelioid cells in CSF, elevated opening pressures, and MRI findings, her neurological findings were consistent with leptomeningeal disease. The absence of malignant cells was likely due to the low sensitivity of cytology. Because of the patient’s otherwise good performance status and limited systemic disease, she was started on weekly intrathecal methotrexate 12 mg mixed with hydrocortisone 25 mg (IT MTX). The patient was continued on oral dexamethasone. Given the progression of disease in the CNS on first-line treatment, she was also switched to second-line systemic therapy with 5-fluorouracil, leucovorin, and irinotecan (FOLFIRI).

The patient had dramatic improvements in her neurological symptoms after the initiation of IT MTX. She had no further seizures, and her headaches and dizziness significantly improved. CEA decreased from 31.9 to 25.5 ng/mL, and CA 19-9 decreased from 7300 to 3522 units/mL. Due to clinical improvement after three months of treatment, the interval of IT MTX was decreased to once every two weeks. In addition, the patient was continued on an extended dexamethasone taper. At the time of writing, seven months after her diagnosis of leptomeningeal disease, the patient reports resolution of dizziness and headaches, as well as improvement in her visual changes and hearing. Currently, she continues on treatment with IT MTX and FOLFIRI once every two weeks. The most recent brain MRI showed persistent but decreased enhancement along the optic sheaths, and LP cytology remains negative.

## Discussion

Leptomeningeal carcinomatosis, also known as leptomeningeal disease, occurs in approximately 5% of all patients with cancer. It is most commonly seen in patients with metastatic breast cancer (5-8%), lung cancer (9-25%), and melanomas (30%) [3]. Malignant cells reach the CNS via either the bloodstream or direct invasion and then spread through the cerebrospinal fluid (CSF) [1]. The most common presenting symptom is pain (76%), including radicular discomfort (58%), headache (32%), or neck/back pain (17%). Other common symptoms are altered mental status (49%), weakness (47%), or seizures (14%) [4].

Leptomeningeal disease in gastric cancer is extremely rare. The prevalence is estimated to be between 0.16% and 0.19% of gastric cancers [5]. Case reports have suggested that the prognosis is poor, with a median survival of two to four months [6,7]. Definitive diagnosis is made by the presence of malignant cells on cytology, although 10% of patients have persistently negative cytology due to low sensitivity [1].

In this case report, the patient had increasing tumor markers despite no objective evidence of systemic progressive disease seen on CT and PET scans. It is likely that her leptomeningeal disease was responsible for this increase, with the CNS serving as a sanctuary site of spread. It is important to consider leptomeningeal disease in cases where tumor markers are increasing in the setting of new-onset neurological symptoms.

There is no established standard of care for leptomeningeal disease in gastric cancer. Table 1 compares treatments and outcomes between this case report and previous case reports, which shows widely varying treatments with survival ranging from two to eleven months. Treatment options are limited as many systemic therapies do not adequately penetrate the blood-brain barrier. However, studies of leptomeningeal disease in breast cancer have shown that chemoradiotherapy is superior to chemotherapy or radiation therapy alone [7]. A case report has documented successful treatment with IT MTX, temozolomide, and radiotherapy [8]. Our patient responded well to IT MTX and continues on therapy seven months after the diagnosis of leptomeningeal disease, in comparison to the median survival of two to four months.

**Table 1:** Selected literature cases of leptomeningeal disease in gastric cancer. MRI: magnetic resonance imaging, CSF: cerebrospinal fluid, IT: intrathecal.

Reference	Year	Patient age and sex	Histology	MRI findings	CSF findings	Treatment	Survival after diagnosis of leptomeningeal disease (months)
This case report	2022	63 F	Poorly differentiated	Abnormal enhancement along bilateral optic nerve sheathes	Atypical epithelioid cells	IT methotrexate; systemic irinotecan, 5-fluorouracil, leucovorin	>7 (Still alive)
Liu [2]	2017	56 M	Poorly differentiated	Negative	Malignant cells	IT methotrexate; radiation; systemic 5-fluorouracil, leucovorin, oxaliplatin	11 (Died in car accident)
Asterita et al [7]	2016	48 F	Signet ring cell	Leptomeningeal enhancement, malignant infiltrates in the frontal lobe and cerebellum	Malignant cells	IT methotrexate; systemic 5-fluorouracil, folic acid, oxaliplatin, docetaxel	2
Kim et al. [6]	2014	37 M	Signet ring cell	Sulcus enhancement	Malignant cells	Supportive	4
Kim et al. [6]	2014	67 M	Moderately differentiated	Negative	Malignant cells	Radiation	2
Kim et al. [6]	2014	47 M	Undifferentiated	Diffuse brain and spinal cord leptomeningeal carcinomatosis	Negative	Radiation; systemic 5-fluorouracil, cisplatin	10
Kim et al. [6]	2014	64 F	Moderately differentiated	Leptomeningeal enhancement	Malignant cells	Radiation	3
Kim et al. [6]	2014	65 M	Moderately differentiated	Leptomeningeal enhancement	Malignant cells	Supportive	1
Kim et al. [6]	2014	72 F	Poorly differentiated with signet ring cells	Leptomeningeal enhancement	Malignant cells	Radiation	4
Kim et al. [6]	2014	42 M	Poorly differentiated	Sulcus enhancement	Malignant cells	Supportive	1
Kim et al. [6]	2014	53 M	Poorly differentiated	Sulcus enhancement	Malignant cells	IT methotrexate	2
Kim et al. [6]	2014	53 M	Poorly differentiated	Ventricle enhancement	Malignant cells	IT methotrexate	2

## Conclusions

Leptomeningeal disease should be considered with increasing tumor markers and a lack of evidence for systemic progression, particularly in patients with new neurological symptoms. The treatment for the leptomeningeal disease in metastatic gastric cancer is not well studied due to the rarity of the condition. Treatment options are limited due to the inability of many medications to penetrate the blood-brain barrier. In this report, we presented a case of leptomeningeal disease in a patient with metastatic gastric cancer who responded well to treatment with IT MTX. Therefore, we propose IT MTX as a potential treatment for patients with leptomeningeal disease in gastric cancer with a preserved functional status. However, further research is needed to evaluate the optimal frequency and length of therapy.

## References

[1] Grossman SA, Krabak MJ (1999). Leptomeningeal carcinomatosis. Cancer Treat Rev.

[2] Liu Y (2017). Leptomeningeal carcinomatosis from gastric cancer successfully treated by the intrathecal methotrexate plus temozolomide and simultaneous radiotherapy: case report and literatures review. Cancer Biol Ther.

[3] El Shafie RA, Böhm K, Weber D (2019). Outcome and prognostic factors following palliative craniospinal irradiation for leptomeningeal carcinomatosis. Cancer Manag Res.

[4] Kaplan JG, DeSouza TG, Farkash A (1990). Leptomeningeal metastases: comparison of clinical features and laboratory data of solid tumors, lymphomas and leukemias. J Neurooncol.

[5] Vergoulidou M (2017). Leptomeningeal carcinomatosis in gastric cancer: a therapeutical challenge. Biomark Insights.

[6] Kim NH, Kim JH, Chin HM, Jun KH (2014). Leptomeningeal carcinomatosis from gastric cancer: single institute retrospective analysis of 9 cases. Ann Surg Treat Res.

[7] Asterita R, Chandrasekar V, Gill D (2016). Gastric cancer with leptomeningeal carcinomatosis: rare but deadly. Am J Gastroenterol.

[8] Kak M, Nanda R, Ramsdale EE, Lukas RV (2015). Treatment of leptomeningeal carcinomatosis: current challenges and future opportunities. J Clin Neurosci.

